# Early rheumatoid arthritis is characterised by a distinct and transient synovial fluid cytokine profile of T cell and stromal cell origin

**DOI:** 10.1186/s13075-019-2026-4

**Published:** 2019-11-06

**Authors:** K. Raza

**Affiliations:** 1 Research into Inflammatory Arthritis Centre Versus Arthritis, and MRC-Versus Arthritis Centre for Musculoskeletal Ageing Research, Institute of Inflammation and Ageing (IIA), University of Birmingham, Queen Elizabeth Hospital, Birmingham, UK; 2 Department of Rheumatology, Sandwell and West Birmingham Hospitals NHS Trust, Birmingham, UK

**Keywords:** Rheumatoid arthritis, Early arthritis, Cytokines, Fibroblasts

## Abstract

A study by Raza et al., published in this journal in 2005, identified that RA patients, within 3 months of symptom onset, had a synovial fluid cytokine profile that was distinct from that of patients with other inflammatory arthritides of similarly short duration. This profile, which was transient, was characterised by cytokines of stromal and T cell origin. These findings suggested that the first few months after symptom onset were associated with changes in the early RA joint that differed from those operating at later stages. The significance of this paper’s methodological approach and its findings, and how they relate to subsequent literature, are discussed.

The importance of the early introduction of disease-modifying anti-rheumatic drug (DMARD) therapies for patients with rheumatoid arthritis (RA) has been widely recognised for over 25 years [1, 2]. Justifications for this position in the 1990s included “there is nothing to be gained by waiting” and that “long term outcome may be altered” [1]. Both of these are now widely held to be correct. However, key issues that remained unclear in the 1990s and early 2000s were as follows: (i) Why does early treatment lead to improved outcomes? Specifically, are disease processes operating in patients with early synovial inflammation qualitatively different from those operating in the joints of patients with longer standing RA, and thus more tractable to treatment. (ii) If early treatment is important, how can we predict the development of RA in patients with newly presenting synovitis, thus helping us to target DMARDs to appropriate individuals? (iii) How time-limited is this “window of opportunity”?

In the early 2000s, the rheumatology group in Birmingham, UK, established an inception cohort of patients with newly presenting clinically apparent synovitis to address some of these issues. A key element of this endeavour was the collection of synovial fluid from recently inflamed joints to study their cellular and cytokine compositions [3–5]. Using a multiplex detection system, we assessed a panel of 23 cytokines and chemokines in synovial fluid from 36 patients with non-crystal-related inflammatory arthritis, of 3-month duration or less, whose final diagnoses were determined after 18-month follow-up [5]. Importantly, we were very clear about our definition of disease duration, timing it from when the patient first reported inflammatory type joint pain and/or early morning stiffness and/or joint-related soft tissue swelling. We reported that the levels of a range of T cell, macrophage and stromal cell-related cytokines (e.g. IL-2, IL-4, IL-13, IL-17, IL-15, basic fibroblast growth factor and epidermal growth factor) were significantly elevated in the synovial fluid of early RA patients, as compared with early arthritis patients who did not develop RA [5]. Other cytokines such as IL-6 did not distinguish between different outcome groups, suggesting their importance in synovitis per se rather than a specific role in rheumatoid synovitis. The transient nature of this early RA-associated cytokine profile was suggested by the fact that it was not present in patients with established RA nor from patients with *early* RA who had further synovial fluid samples collected after the first 3 months of symptoms [5].

This was one of the first studies to suggest that the first few months after the onset of symptoms may be associated with pathological changes in the early RA joint that differed from those operating at later stages—providing a potential explanation for the differential response to DMARDs in patients with early RA compared to longer standing disease. Whilst this study was not able to address the issue of the duration of this window, subsequent work has suggested that the first 3 to 4 months after symptom onset does represent an important therapeutic window in patients with RA [6, 7].

Our study had a number of important limitations including the fact that we were not able to study synovial tissue and compare that with synovial fluid. The subsequent development of minimally invasive ultrasound-guided synovial biopsy techniques has allowed us, and others, to access synovial tissue from early arthritis patients. The concept that pathological processes operating during the first 3 months of symptoms differ from those during later stages has now been supported by findings that CXCL4 and CXCL7 are transiently increased in the synovium of early RA patients [8]. Fibroblasts have long been recognised to play a key role in driving the persistence of inflammation in patients with RA [9]. Our findings related to stromal cytokines suggested that fibroblasts may play a particular role during the establishment of joint inflammation in early RA. This has been supported by data showing that the stromal marker fibroblast activation protein (FAP) is elevated in patients with early RA compared with other early arthritis groups [10] and that synovial fibroblasts from RA patients with a short duration of synovitis exhibit a transient functional phenotype that contributes to the accumulation of persistent infiltrates [11]. Furthermore, it has been shown that the formation of tertiary lymphoid structures, seen in a subset of patients with RA, is regulated by FAP-positive stromal cells, in a manner that is dependent upon the autocrine and paracrine production of IL-13 [12]— a cytokine we identified in the synovial fluid of patients with early RA.

Since the publication of our initial findings in 2005, there has been increased focus on the earliest phases of RA including the stages preceding the onset of joint swelling [13]. Whilst we were not able to study these, a clearer understanding of these earliest phases and how to define them [14–16] now opens the possibility of assessing when, during the natural history of RA, synovial pathology first develops and how this changes over time. In the context of this, an important prospective study by de Hair and colleagues assessed the synovium in seropositive individuals without clinical arthritis [17]. In most individuals, there was no significant subclinical synovitis and no clear association between the presence of inflammatory cells and subsequent development of arthritis, although there was a trend towards an association between synovial CD3+ T cell numbers and later progression to arthritis [17]. The availability of new methodologies, including single-cell RNA sequencing, is beginning to shed important new light on disease processes operating in the joints of patients with established RA [18]. Applying these technologies to the study of synovium from carefully phenotyped patients with, and at risk of, RA in whom disease durations are captured in a standardised way [16] should shed new light on processes driving the establishment of RA as a joint centric disease.

## Data Availability

Not applicable.
